# Catalytic Performance of AAEM-Loaded Biochars for Regulating Anhydrosugar Formation During Cellulose Pyrolysis

**DOI:** 10.3390/nano16160983

**Published:** 2026-08-10

**Authors:** Guang Hu, Tingting Zhou, Yuxin Wei, Kuankuan Liu, Jing Tang, Junqi Wang

**Affiliations:** 1 School of Nuclear Science and Technology, Xi’an Jiaotong University, Xi’an 710049, China; guanghu@xjtu.edu.cn; 2 School of Human Settlements and Civil Engineering, Xi’an Jiaotong University, Xi’an 710049, China; 372498563@stu.xjtu.edu.cn (T.Z.); weiyuxin5924@stu.xjtu.edu.cn (Y.W.); lk20010422@stu.xjtu.edu.cn (K.L.); j.tang@stu.xjtu.edu.cn (J.T.)

**Keywords:** cellulose pyrolysis, biochar catalyst, alkali and alkaline earth metals (AAEM), levoglucosan (LG), levoglucosenone (LGO), kinetic analysis

## Abstract

Biochar has attracted increasing attention as a low-cost catalyst for biomass pyrolysis due to its developed pore structure, abundant surface functional groups and tunable physicochemical properties. In this study, biochars loaded with alkali and alkaline earth metal (AAEM) species were prepared by pyrolyzing cellulose impregnated with different chloride and acetate salts, including NaCl, KCl, CaCl_2_, MgCl_2_, CH_3_COONa, CH_3_COOK, (CH_3_COO)_2_Ca and (CH_3_COO)_2_Mg. The resulting AAEM-loaded biochars were subsequently employed as catalysts for cellulose pyrolysis to investigate their effects on product distribution, particularly levoglucosan (LG) and levoglucosenone (LGO) formation. SEM and XRD analyses revealed that the AAEM precursor significantly affected the morphology and phase composition of the biochars. Chloride-derived biochars retained crystalline salt phases or formed corresponding metal oxides, whereas acetate-derived biochars exhibited more dispersed metal-containing species. The introduction of AAEM-loaded biochars generally decreased bio-oil and LG yields while increasing char production, indicating enhanced secondary cracking and repolymerization reactions. Among the investigated catalysts, alkali metal-loaded biochars exhibited stronger inhibition toward LG formation than alkaline earth metal-loaded biochars. The catalytic effects followed the order of C-KCl ≈ C-NaCl > C-MgCl_2_ > C-CaCl_2_ for chloride-derived biochars and C-CH_3_COOK ≈ C-CH_3_COONa > C-(CH_3_COO)_2_Mg > C-(CH_3_COO)_2_Ca for acetate-derived biochars. Notably, C-(CH_3_COO)_2_Ca and C-(CH_3_COO)_2_Mg slightly promoted LGO formation, which was attributed to the synergistic effects of alkaline earth metal species, surface oxygen-containing functional groups and acetate-derived intermediates on dehydration reactions. Thermogravimetric and kinetic analyses further demonstrated that AAEM-loaded biochars reduced the apparent activation energy of cellulose pyrolysis and facilitated thermal decomposition. These findings provide new insights into the catalytic role of AAEM-loaded biochars and suggest a promising strategy for regulating anhydrosugar selectivity, particularly for the production of high-value LGO from biomass pyrolysis.

## 1. Introduction

Pyrolysis of lignocellulosic biomass can generally be classified into slow, intermediate, fast, and flash pyrolysis according to the heating rate and residence time. Slow pyrolysis mainly favors biochar production [1], whereas intermediate pyrolysis generally results in a more balanced distribution of solid, liquid, and gaseous products [2]. Fast and flash pyrolysis involve more rapid heating and shorter vapor residence times, thereby favoring the release and recovery of volatile and condensable products [3,4]. The selection of a pyrolysis mode therefore depends on the desired product and research objective. In the present study, rapid-heating pyrolysis was employed because the investigation focused on cellulose-derived condensable products, particularly levoglucosan (LG) and levoglucosenone (LGO), which are important platform chemicals with applications in pharmaceuticals, polymers, and fine chemical synthesis [5,6,7]. However, the formation of LG and LGO is highly dependent on pyrolysis conditions and catalytic environments [8,9]. Therefore, developing efficient catalysts for regulating cellulose pyrolysis pathways and improving product selectivity remains an important research topic [10,11].

Biochar, the major solid product generated during biomass pyrolysis, possesses abundant oxygen-containing functional groups, developed pore structures, and relatively large specific surface areas [12,13,14]. These characteristics make biochar an attractive low-cost catalyst or catalyst support for biomass conversion processes. Previous studies have demonstrated that biochar can significantly influence the distribution of pyrolysis products. Liu et al. [15]. reported that the addition of cotton-straw-derived biochar promoted phenol formation during pyrolysis, increasing the phenol yield from 30.15 wt% to 45.25 wt% at a biomass-to-biochar ratio of 1:1. Ren et al. [16]. investigated the catalytic pyrolysis of pinewood over corncob biochar and found that biochar promoted the formation of phenolic and hydrocarbon compounds while suppressing primary pyrolysis reactions. These studies indicate that biochar can effectively regulate secondary conversion reactions of pyrolysis vapors and alter the selectivity of target products.

In addition to the intrinsic properties of biochar, metal loading can further modify its catalytic performance. Wang et al. [17]. found that NaCl- and FeCl_3_-loaded biochars promoted the cracking of oxygen-containing compounds and unsaturated side chains in bio-oil, resulting in enhanced phenol production. In contrast, MgCl_2_-loaded biochar decreased the content of acetol while increasing the formation of cyclopentanone derivatives. Furthermore, MgO-modified biochars have been reported to promote the formation of furans and ketones, including LGO, during biomass pyrolysis [18]. These findings suggest that metal species play a critical role in determining the catalytic behavior of biochar and the distribution of pyrolysis products. Alkali and alkaline earth metals (AAEMs), such as Na, K, Ca and Mg, are widely recognized as important catalytic species during biomass pyrolysis [19,20,21]. Numerous studies have demonstrated that AAEMs can promote dehydration, fragmentation and secondary conversion reactions of cellulose-derived intermediates, thereby significantly affecting the formation of anhydrosugars [22,23]. Nevertheless, most existing studies have focused on the direct addition of AAEM salts, whereas the catalytic behavior of AAEM-containing biochars generated from AAEM-impregnated biomass has received much less attention. Such biochars combine the porous carbon matrix with AAEM-derived active species and may provide a unique catalytic environment for regulating cellulose pyrolysis pathways.

Therefore, in this work, biochars prepared from cellulose impregnated with different AAEM salts were employed as catalysts for cellulose pyrolysis. Microcrystalline cellulose was selected as the biochar precursor because of its well-defined composition and its direct relevance to anhydrosugar formation. Compared with raw lignocellulosic biomass, the use of cellulose minimizes the interference of lignin, hemicellulose, extractives and inherent mineral species, thereby allowing the catalytic effects of the introduced AAEM species to be evaluated more clearly. The physicochemical properties of the resulting AAEM-loaded biochars were characterized. Their effects on product distribution, particularly the formation of LG and LGO, were systematically investigated through pyrolysis experiments, thermogravimetric analysis and kinetic calculations. The results provide new insights into the catalytic role of AAEM-loaded biochars and offer a potential strategy for regulating anhydrosugar formation during biomass pyrolysis.

## 2. Method

### 2.1. Materials

Microcrystalline cellulose powder (100–200 mesh, 43.2 wt% C, 6.2 wt% H and 50.1 wt% O) was purchased from Shanghai Yuanye Bio-Technology Co., Ltd. (Shanghai, China). Potassium chloride (KCl), sodium chloride (NaCl), calcium chloride (CaCl_2_), magnesium chloride (MgCl_2_), potassium acetate (CH_3_COOK), sodium acetate (CH_3_COONa), calcium acetate ((CH_3_COO)_2_ Ca), and magnesium acetate ((CH_3_COO)_2_Mg) were obtained from Aladdin Reagent Co., Ltd. (Shanghai, China). All chemicals were of analytical grade and used without further purification.

AAEM-loaded cellulose samples were prepared by impregnating microcrystalline cellulose with aqueous solutions of KCl, NaCl, CaCl_2_, MgCl_2_, CH_3_COOK, CH_3_COONa, (CH_3_COO)_2_Ca, and (CH_3_COO)_2_Mg at loading levels of 1 wt% and 5 wt%. After shaking for 24 h and drying at 105 °C for 12 h, the impregnated samples were pyrolyzed under an N_2_ atmosphere to obtain AAEM-loaded biochars, which were subsequently used as catalysts for cellulose pyrolysis.

### 2.2. Pyrolysis Experiments

Catalytic pyrolysis experiments were conducted in a fixed-bed tubular reactor (OTF-1200X, Kejing, Hefei, China). Before each experiment, the biochar catalyst was mixed with microcrystalline cellulose at a catalyst loading of 3 wt%, corresponding to a cellulose/catalyst mass ratio of 100:3. The samples were pyrolyzed at 540 °C for 145 s under a continuous N_2_ flow. The volatile products generated during pyrolysis were rapidly condensed and collected using a series of gas–liquid separators, while the non-condensable gases were discharged for subsequent analysis. All catalytic pyrolysis experiments were independently performed in triplicate, and the product yields are reported as the mean ± standard deviation. The experimental setup is based on our previous experiments [24].

**Figure 1:**
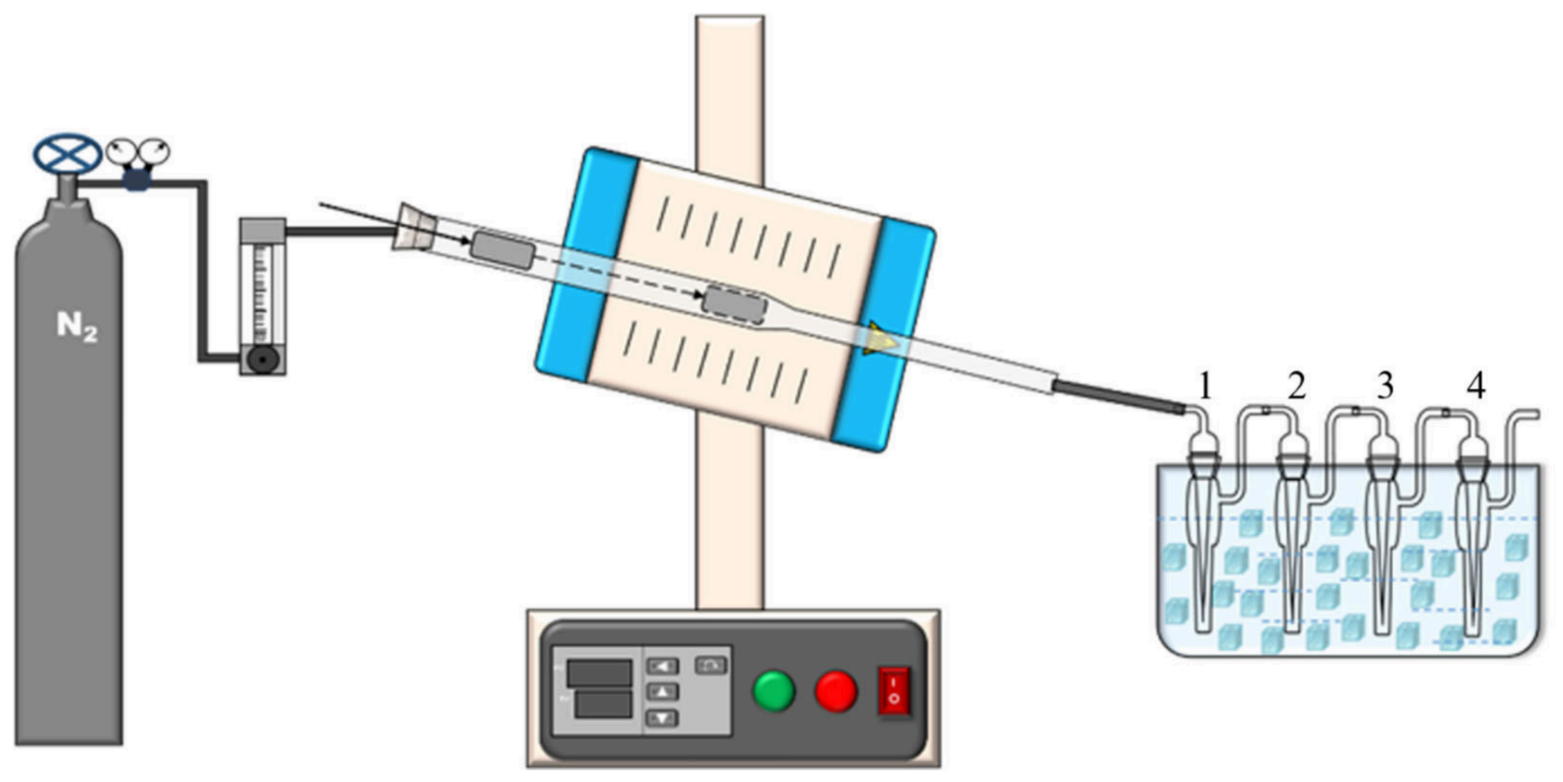
Pyrolysis device diagram.

Biochar and bio-oil yields were determined gravimetrically from the recovered solid residue and the net mass increase in the first two condensation bottles, respectively. The bio-oil collected in the first two bottles was combined and mixed with 16 mL of the chloroform/methanol trapping solution (4:1, *v*/*v*) collected from the third and fourth bottles (Figure 1). The resulting solution was filtered through a 0.22 μm membrane before GC analysis. LG and LGO were identified by comparison with authentic standards and quantified using the external standard method on a GC-2010 Pro gas chromatograph equipped with an Elite-WAX column (30 m × 0.25 mm × 0.25 μm). The injector temperature was 230 °C, and N_2_ was used as the carrier gas at 2 mL min^−1^. The oven temperature was maintained at 50 °C for 2 min, increased to 230 °C at 10 °C min^−1^, and held for 29 min. To evaluate the influence of AAEM-loaded biochars on cellulose pyrolysis behavior, thermogravimetric analysis (TGA) was carried out using a STA449F5 thermal analyzer (Netzsch, Selb, Germany). Approximately 6 mg of sample was heated from 30 to 650 °C under an N_2_ atmosphere at heating rates of 10 °C min^−1^.

### 2.3. Characterization of AAEM-Loaded Biochars

The morphology and elemental distribution of the AAEM-loaded biochars were investigated using scanning electron microscopy coupled with energy-dispersive spectroscopy (SEM-EDS, GeminiSEM 500, Zeiss, Jena, Germany). The surface structure evolution induced by different AAEM species was comparatively analyzed.

The crystalline structure and phase composition of the biochars were characterized by X-ray diffraction (XRD, XRD-6100, Shimadzu, Kyoto, Japan) using Cu Kα radiation operated at 40 kV and 30 mA. Diffraction patterns were collected over a 2θ range of 10–80° to evaluate the effects of AAEM impregnation on the carbon structure and mineral phases.

## 3. Discussion

### 3.1. SEM of AAEM-Loaded Biochars

**Figure 2:**
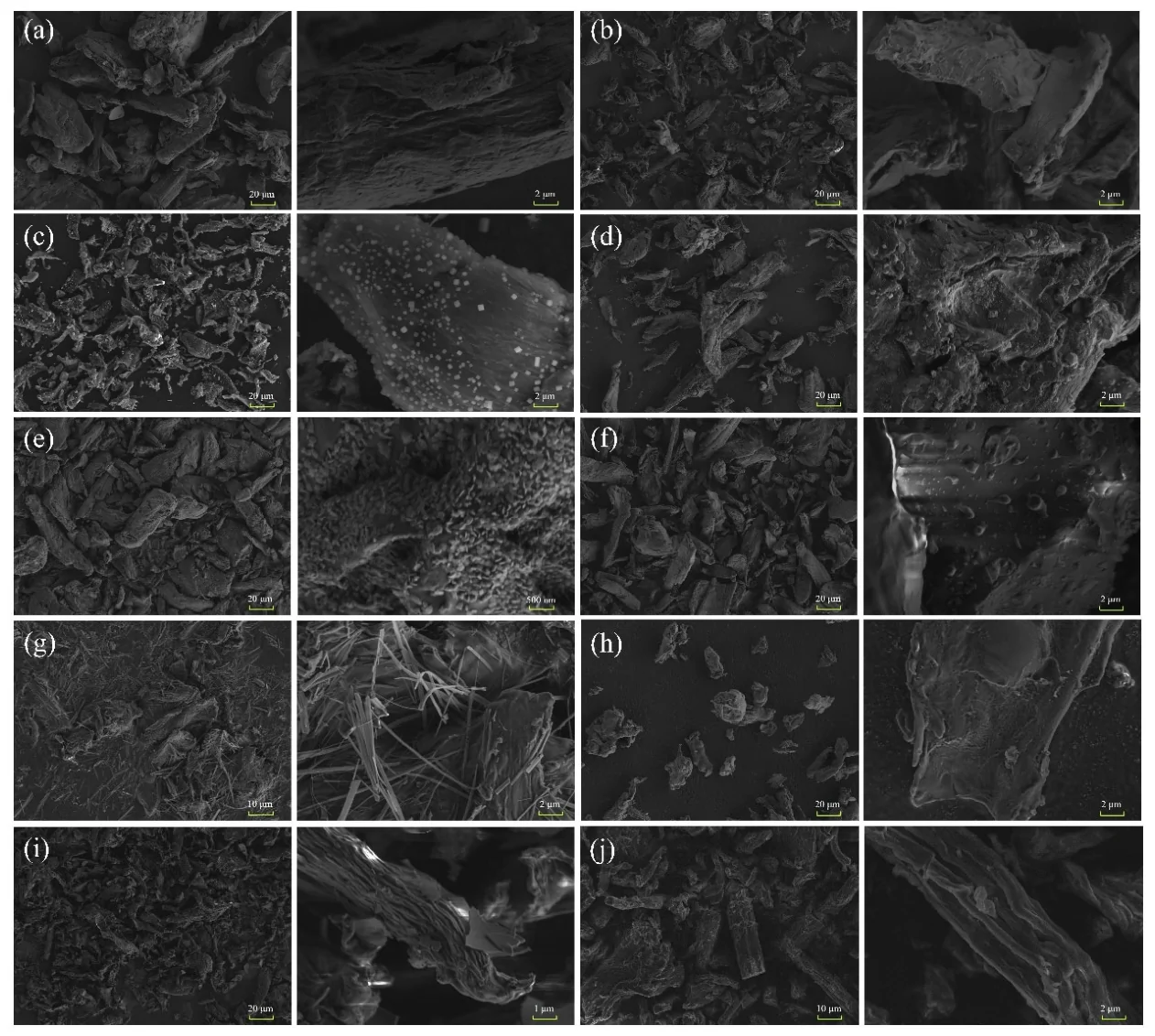
SEM images of pristine biochar and modified biochars. (**a**) Pristine cellulose-derived biochar, (**b**) pyrolyzed biochar, (**c**) C-NaCl, (**d**) C-KCl, (**e**) C-CaCl_2_, (**f**) C-MgCl_2_, (**g**) C-CH_3_COONa, (**h**) C-CH_3_COOK, (**i**) C-(CH_3_COO)_2_ Ca, (**j**) C-(CH_3_COO)_2_ Mg.

SEM images revealed substantial changes in the morphology of biochars after AAEM impregnation and pyrolysis (Figure 2). The pristine cellulose-derived biochar retained the rod-like morphology inherited from the original cellulose particles, exhibiting a relatively rough surface with wrinkles and limited pore development. Such a morphology is typical of cellulose chars produced through devolatilization and carbonization processes, during which the ordered cellulose structure is gradually transformed into disordered carbonaceous frameworks.

After AAEM impregnation, distinct surface features were observed depending on the type of salt employed. For chloride-derived biochars, numerous inorganic particles were distributed on the carbon surface. In particular, C-NaCl and C-KCl exhibited abundant angular crystalline particles, indicating that these salts largely preserved their crystalline structures during pyrolysis. EDS analysis further confirmed the presence of Na, K and Cl species on the biochar surface. In contrast, C-CaCl_2_ and C-MgCl_2_ displayed densely distributed fine particles and a rougher surface texture. Combined with EDS and XRD results, these particles were attributed to CaO and MgO generated through the thermal decomposition of the corresponding chloride salts during pyrolysis. Compared with chloride salts, acetate-derived biochars exhibited a more pronounced structural evolution. Biochars prepared from CH_3_COONa and CH_3_COOK showed highly wrinkled surfaces accompanied by filamentous or network-like structures, while C-(CH_3_COO)_2_ Ca and C-(CH_3_COO)_2_ Mg contained abundant sheet-like or finely dispersed particles. The decomposition of acetate anions during pyrolysis likely promoted the redistribution of metal species and generated a more heterogeneous surface structure. Such structural features may increase the contact probability between volatile intermediates and catalytic sites, thereby enhancing secondary cracking, dehydration and reforming reactions during cellulose pyrolysis.

Overall, all AAEM-loaded biochars maintained the basic carbon skeleton of cellulose char while developing distinct surface morphologies depending on the metal species and precursor salts. The rough surfaces, wrinkles, and dispersed inorganic phases observed on the AAEM-loaded biochars are expected to provide additional active sites for interactions with pyrolysis vapors, which may contribute to their catalytic performance during cellulose pyrolysis.

### 3.2. XRD of AAEM-Loaded Biochars

**Figure 3:**
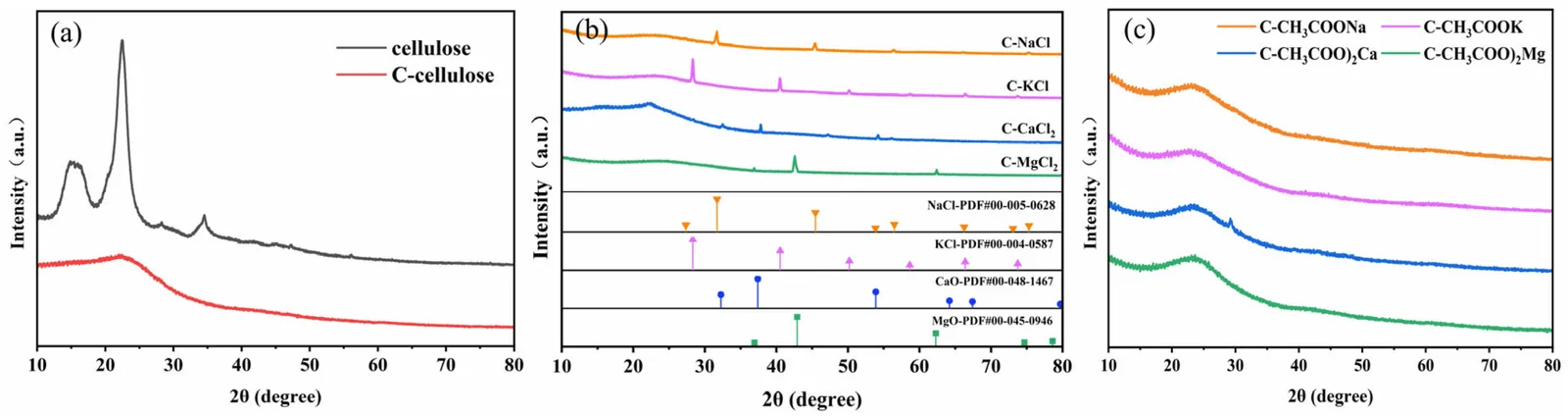
XRD patterns of (**a**) pristine cellulose-derived biochar and pyrolyzed biochar, (**b**) chloride-derived biochars, and (**c**) acetate-derived biochars.

The crystalline structures of cellulose, cellulose-derived biochar, and AAEM-loaded biochars were investigated by XRD (Figure 3). Microcrystalline cellulose exhibited characteristic diffraction peaks at approximately 16° and 22°, corresponding to the cellulose I structure. After pyrolysis at 540 °C, these characteristic peaks were no longer discernible, indicating the destruction of the ordered cellulose crystalline structure. The cellulose-derived biochar exhibited a broad diffraction band centered at approximately 23°, corresponding to the (002) plane of poorly ordered carbon. The broad diffraction background indicates that the biochar predominantly consisted of poorly ordered aromatic carbon rather than highly graphitized carbon, consistent with the aromatization and carbonization of cellulose during pyrolysis.

For chloride-derived biochars, additional crystalline phases were detected. C-NaCl and C-KCl displayed characteristic diffraction peaks corresponding to crystalline NaCl and KCl, indicating that these alkali metal chlorides remained largely stable under the pyrolysis conditions employed in this study. In contrast, characteristic diffraction peaks assigned to CaO and MgO were observed in C-CaCl_2_ and C-MgCl_2_. This result suggests that alkaline earth metal chlorides underwent thermal transformation during pyrolysis, generating corresponding metal oxides that remained dispersed within the carbon matrix [25,26]. The formation of CaO and MgO is consistent with the SEM observations showing abundant fine particles on the biochar surface. Interestingly, acetate-derived biochars exhibited much weaker crystalline features. Apart from the broad carbon diffraction peaks, no obvious crystalline phases were observed. This phenomenon indicates that the decomposition of acetate salts during pyrolysis resulted in highly dispersed metal species or amorphous inorganic phases rather than large crystalline domains [27]. Such highly dispersed active species are generally considered beneficial for catalytic reactions because they provide a larger accessible surface area and more exposed active sites. Taken together, the XRD results demonstrate that the nature of the AAEM precursor strongly influences the final mineral phase composition of the biochars. While chloride salts tend to preserve crystalline phases or form stable metal oxides, acetate salts favor the generation of highly dispersed or amorphous metal-containing species.

### 3.3. Effects of AAEM-Loaded Biochars on Cellulose Pyrolysis Products

**Figure 4:**
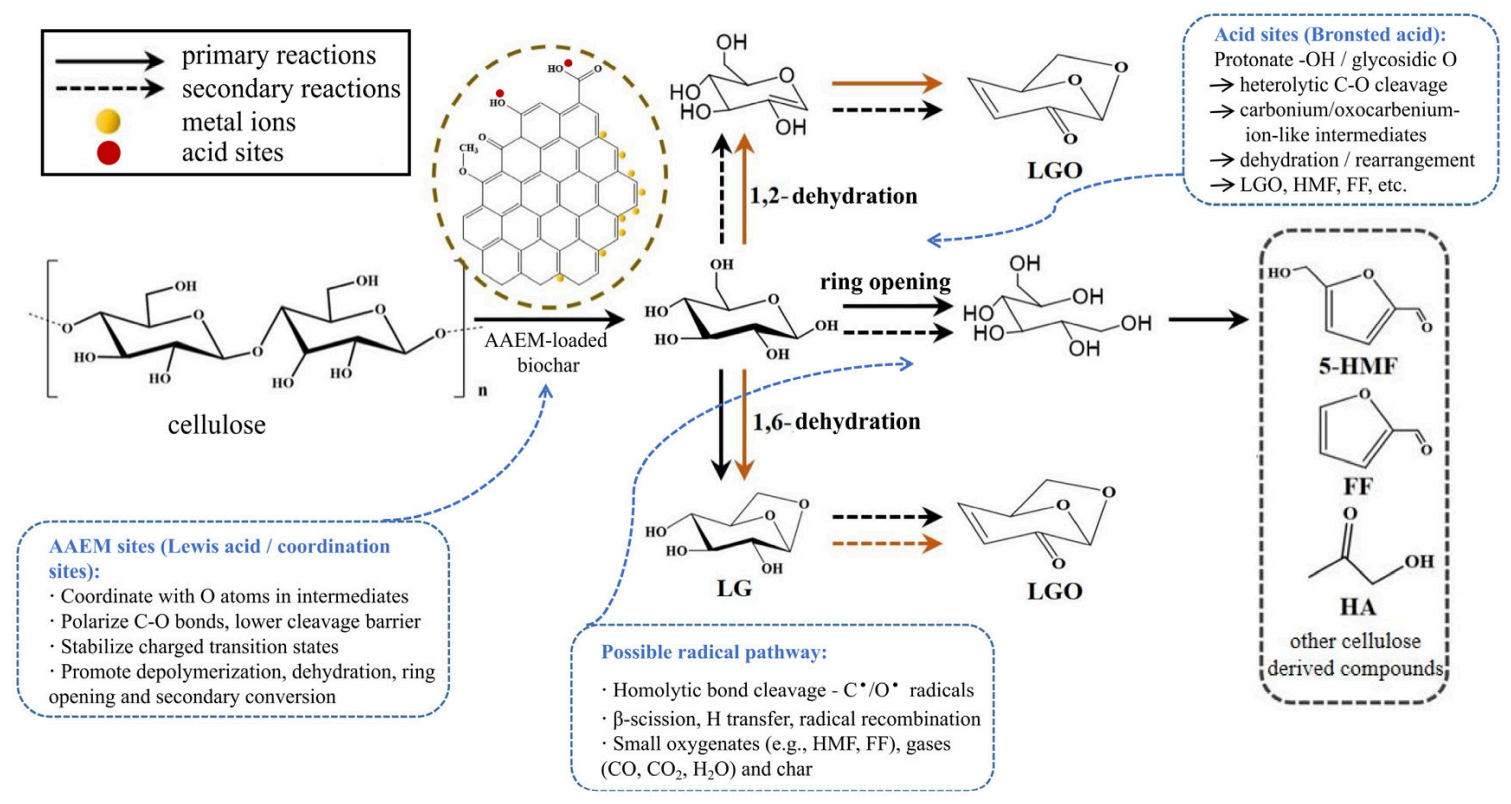
Schematic illustration of the catalytic mechanism of AAEM-loaded biochar for cellulose pyrolysis toward the formation of LGO.

The effects of AAEM-loaded biochars on the distribution of cellulose pyrolysis products are presented in Figure 4. Compared with non-catalytic pyrolysis, the introduction of biochar catalysts generally decreased bio-oil yield while promoting char formation, indicating that secondary reactions of pyrolysis vapors were significantly enhanced on the biochar surface.

For chloride-derived biochars, all catalysts reduced bio-oil production and increased char yield to varying extents. The reductions in bio-oil yield followed the order C-NaCl (12.0 wt%) > C-KCl (9.5 wt%) > C-MgCl_2_ (3.9 wt%) > C-CaCl_2_ (1.6 wt%), whereas the corresponding increases in char yield ranged from 3.7 to 5.7 wt%. These results suggest that the AAEM-loaded biochars promoted secondary cracking, cross-linking and repolymerization reactions of volatile intermediates [28,29]. The porous carbon matrix can absorb condensable pyrolysis vapors, prolonging their residence time within the catalytic environment. Meanwhile, the surface oxygen-containing functional groups and metal species provide active sites for dehydration, decarboxylation and aromatization reactions, resulting in increased char formation at the expense of condensable bio-oil [30] (Figure 4).

The influence of AAEM-loaded biochars was even more evident in the formation of LG and LGO. Relative to the blank biochar, AAEM-loaded biochars exhibited a stronger inhibitory effect on LG formation. In particular, C-NaCl and C-KCl decreased LG yields by 7.1 wt% and 7.0 wt%, respectively, whereas C-CaCl_2_ and C-MgCl_2_ reduced LG yields by 5.7 wt% and 4.7 wt%. This observation indicates that alkali metal-containing biochars were more effective than alkaline earth metal-loaded biochars in suppressing depolymerization pathways leading to LG formation. The stronger catalytic effect of Na- and K-loaded biochars can be attributed to the higher dispersion of alkali metal species on the carbon surface. SEM observations showed that C-NaCl and C-KCl contained finer inorganic particles than C-CaCl_2_ and C-MgCl_2_, providing greater contact opportunities between catalytic sites and volatile intermediates. Furthermore, alkali metals can interact strongly with hydroxyl groups in cellulose-derived intermediates, facilitating ring-opening, fragmentation and dehydration reactions. As a result, the reaction pathway shifts away from glycosidic bond cleavage and LG formation toward the generation of smaller oxygenated compounds and secondary char precursors (Figure 4).

**Figure 5:**
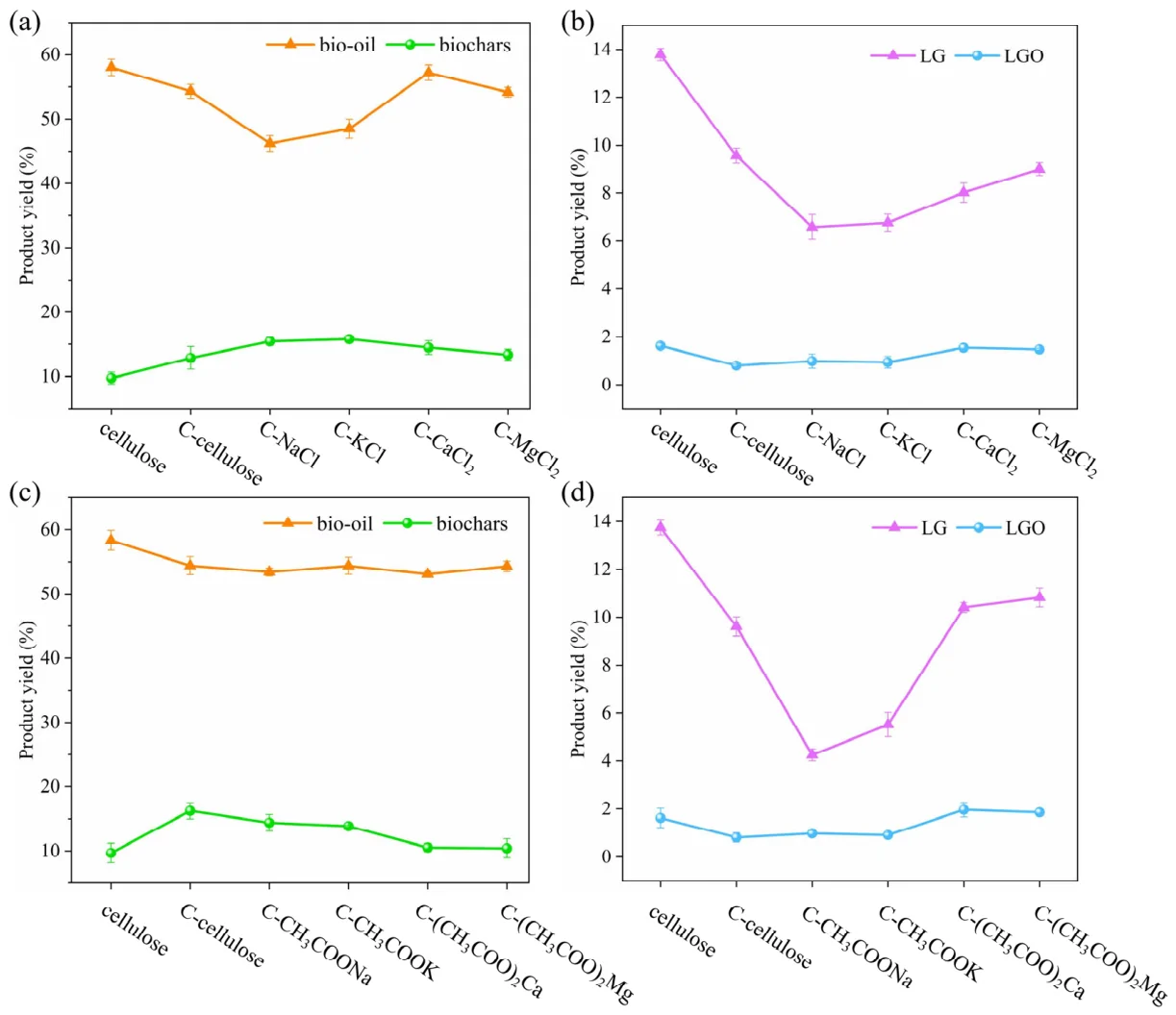
Effects of chloride- and acetate- derived biochars on the product yields from cellulose pyrolysis: (**a**) yields of bio-oil and biochar over chloride-derived biochars, (**b**) yields of LG and LGO over chloride-derived biochars, (**c**) yields of bio-oil and biochar over acetate-derived biochars, (**d**) yields of LG and LGO over acetate-derived biochars.

In contrast, acetate-derived biochars exerted a weaker influence on the overall product distribution (Figure 5). Although all acetate-derived biochars decreased bio-oil yields and increased char production, the magnitude of these changes was smaller than that observed for chloride-derived biochars. This suggests that the catalytic activity of acetate-derived biochars toward secondary vapor conversion was relatively limited. Despite their weaker effect on bulk product yields, acetate-derived biochars significantly influenced LG and LGO formation. C-CH_3_COONa and C-CH_3_COOK decreased LG yields by 9.5 wt% and 8.2 wt%, respectively, showing even stronger inhibition than the corresponding chloride-derived catalysts. In contrast, C-(CH_3_COO)_2_Ca and C-(CH_3_COO)_2_Mg only reduced LG yields by 3.4 wt% and 3.0 wt%, respectively. More importantly, these two alkaline earth metal-containing biochars slightly increased LGO yields, whereas all other catalysts reduced LGO production. This unique behavior may be related to the catalytic environment generated by Ca- and Mg-containing acetate-derived biochars. SEM characterization revealed abundant sheet-like structures and developed surface features on these biochars, which could enhance the adsorption and retention of pyrolysis intermediates. The adsorbed intermediates are subsequently exposed to surface oxygen-containing functional groups and alkaline earth metal species, promoting dehydration reactions associated with LGO formation. In addition, acetate-derived species may facilitate the conversion of LG into LGO through secondary dehydration pathways. Consequently, the competitive relationship between LG formation and LGO generation is altered, leading to a slight increase in LGO yield despite the overall suppression of LG production.

### 3.4. Thermogravimetric Analysis of Cellulose Pyrolysis over AAEM-Loaded Biochars

**Figure 6:**
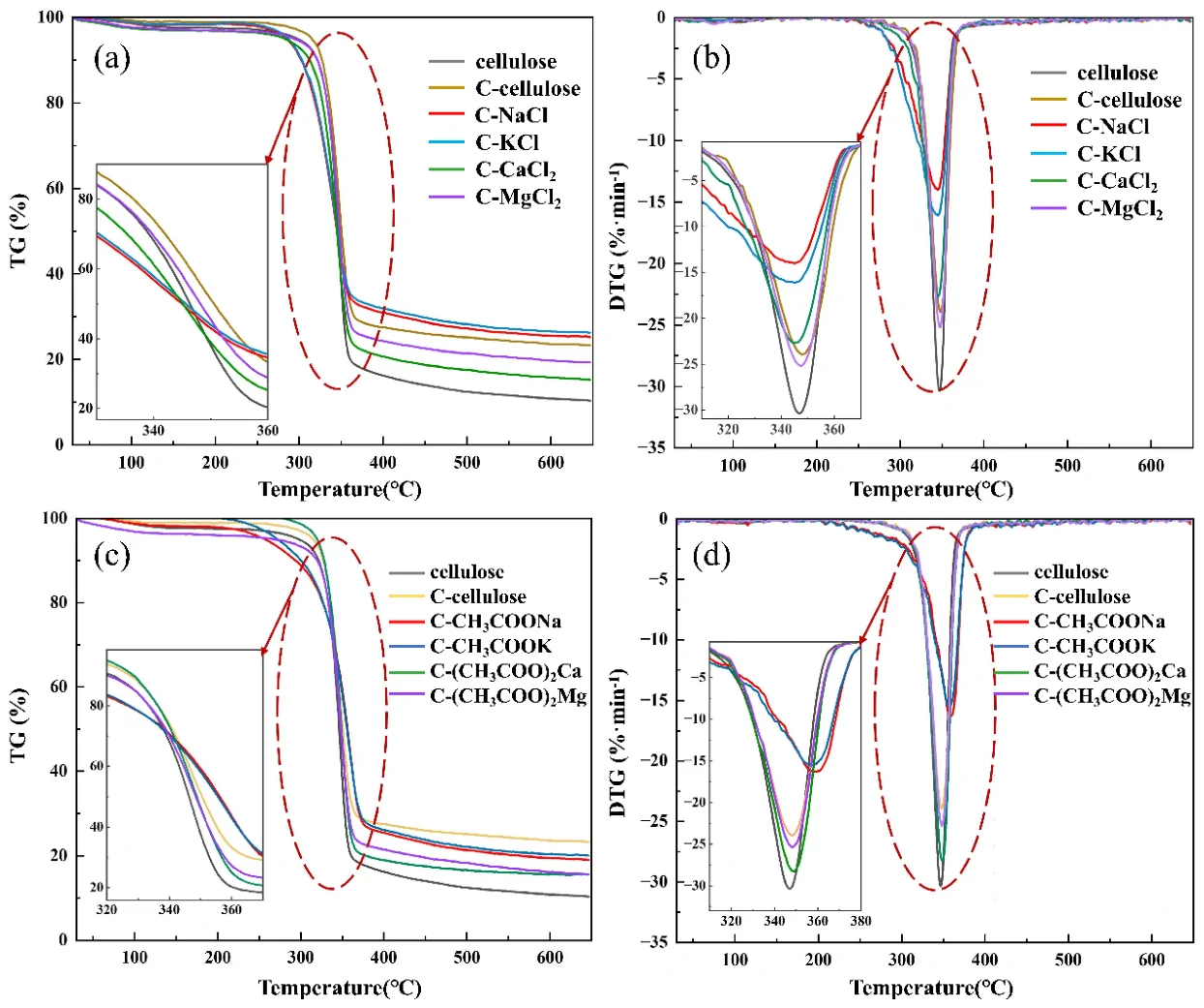
Thermogravimetric analysis of different biochars: (**a**) TG and (**b**) DTG curves of chloride-derived biochars, (**c**) TG and (**d**) DTG curves of acetate-derived biochars.

Thermogravimetric analysis was further performed to clarify the influence of AAEM-loaded biochars on the pyrolysis behavior of cellulose. As shown in Figure 6, the addition of pristine cellulose biochar slightly delayed cellulose decomposition, with the maximum decomposition temperature increasing from 347 °C by approximately 1.1 °C. Meanwhile, the maximum weight-loss rate decreased by 5.4% min^−1^, indicating that the biochar matrix alone mainly acted as a physical barrier and vapor adsorbent rather than an efficient catalytic phase.

In contrast, chloride-derived AAEM-loaded biochars shifted the main decomposition peak toward lower temperatures, suggesting that these catalysts promoted the thermal decomposition of cellulose. C-NaCl, C-KCl, C-CaCl_2_ and C-MgCl_2_ decreased the peak temperature by 6.1, 5.5, 3.4 and 2.6 °C, respectively. The maximum weight-loss rates were also reduced by 14.2, 16.1, 5.2 and 8.3% min^−1^, respectively. This result indicates that AAEM species accelerated the initial cleavage and secondary conversion of cellulose-derived intermediates, but simultaneously weakened the intensity of the main devolatilization stage. Among the chloride-derived biochars, C-NaCl and C-KCl showed stronger effects than C-CaCl_2_ and C-MgCl_2_. This trend is consistent with their stronger suppression of LG formation discussed above. The alkali metal species loaded on biochar can interact with oxygen-containing groups in cellulose-derived intermediates, facilitating dehydration, decarbonylation, decarboxylation and ring-opening reactions. Meanwhile, the rough surface and dispersed salt particles of C-NaCl and C-KCl may increase the contact between volatile intermediates and catalytic sites, promoting secondary cracking and gas formation. Therefore, the decrease in peak temperature reflects catalytic promotion of cellulose decomposition, while the lower maximum decomposition rate suggests that the dominant reaction pathway was diverted from rapid depolymerization toward more gradual catalytic fragmentation and secondary conversion.

For acetate-derived biochars, C-CH_3_COONa and C-CH_3_COOK caused a more pronounced shift in the TG/DTG curves toward lower temperatures. The peak temperature decreased by 10.2 and 10.8 °C, respectively, while the maximum decomposition rate decreased by 15.2 and 14.2% min^−1^. These results demonstrate that Na- and K-containing acetate-derived biochars exhibited the strongest catalytic effect on cellulose pyrolysis. The enhanced activity may be related to the formation of more dispersed alkali metal species during acetate decomposition, which can provide more accessible active sites for the conversion of cellulose-derived volatile intermediates. This explains why C-CH_3_COONa and C-CH_3_COOK markedly inhibited LG formation in the product distribution analysis.

Different from alkali metal acetates, C-(CH_3_COO)_2_Ca and C-(CH_3_COO)_2_Mg slightly increased the peak temperature by 1.2 and 1.1 °C, respectively. However, they still reduced the maximum decomposition rate by 2.8 and 4.5% min^−1^ and lowered the initial decomposition temperature, resulting in a broader main reaction region. This indicates that Ca- and Mg-containing acetate-derived biochars did not simply accelerate cellulose decomposition, but rather broadened the thermal conversion process and promoted secondary transformation of intermediates over a wider temperature range. This behavior is consistent with their ability to promote the conversion of LG toward LGO and other secondary products.

Kinetic analysis further confirmed the catalytic role of AAEM-loaded biochars (Table 1). Compared with pristine biochar, all AAEM-loaded biochars reduced the apparent activation energy of cellulose pyrolysis, indicating a lower energy barrier for thermal decomposition. The catalytic effect followed the order of C-NaCl ≈ C-KCl > C-CaCl_2_ > C-MgCl_2_ for chloride-derived biochars and C-CH_3_COOK ≈ C-CH_3_COONa > C-(CH_3_COO)_2_Ca > C-(CH_3_COO)_2_Mg for acetate-derived biochars. These trends are generally consistent with the observed suppression of LG formation and demonstrate that AAEM species promote cellulose conversion by facilitating bond cleavage and secondary reactions of pyrolysis intermediates.

Overall, the TGA results confirm that the catalytic effect of AAEM-loaded biochars is strongly dependent on both the metal cation and the precursor anion. Na- and K-loaded biochars, especially those derived from acetate salts, significantly lowered the main decomposition temperature and suppressed rapid LG-forming depolymerization pathways. In contrast, Ca- and Mg-loaded, acetate-derived biochars broadened the decomposition region and favored secondary dehydration reactions, which may account for the slight enhancement of LGO formation. These findings further support the conclusion that AAEM-loaded biochars regulate cellulose pyrolysis by altering the balance between depolymerization, dehydration, fragmentation, and secondary vapor conversion pathways.

## 4. Conclusions and Future Work

AAEM-loaded biochars prepared from chloride and acetate salts were characterized and applied as catalysts for cellulose pyrolysis. The introduction of C-AAEM generally decreased bio-oil and LG yields while promoting char formation, indicating enhanced secondary conversion and repolymerization reactions. Among the investigated catalysts, alkali metal-loaded biochars exhibited stronger inhibition toward LG formation than alkaline earth metal-loaded biochars. In contrast, C-(CH_3_COO)_2_Ca and C-(CH_3_COO)_2_Mg slightly promoted LGO formation, which was attributed to the synergistic effects of alkaline earth metals, surface oxygen-containing functional groups, and acetate-derived species on dehydration reactions. TGA and kinetic analyses further confirmed that C-AAEM reduced the apparent activation energy of cellulose pyrolysis and facilitated thermal decomposition. These findings demonstrate that AAEM-loaded biochars can effectively regulate cellulose pyrolysis pathways and provide a promising strategy for the selective production of high-value chemicals, particularly LGO.

It should be noted that the present study primarily focuses on the catalytic effects of AAEM-loaded biochars on cellulose pyrolysis under direct-contact conditions. Because the biochar catalyst was physically mixed with cellulose, the originally added biochar could not be readily separated from the newly formed char after pyrolysis, preventing a reliable evaluation of catalyst stability and recyclability. Future work will employ spatially separated or two-stage pyrolysis systems to enable catalyst recovery and a systematic investigation of the structural stability, deactivation behavior, and reusability of AAEM-loaded biochar catalysts.

## Figures and Tables

**Table 1:** C-AAEM catalytic kinetic parameters for the pyrolysis of cellulose.

Sample	Temperature Range °C	Activation Energy kJ·mol^−1^	Antecedent Factor min^−1^	|*r*|
cellulose	300.4–347.6	157.99	4.93 × 10^10^	0.97
C-cellulose	300.2–345.2	167.88	8.43 × 10^7^	0.98
C-NaCl	278.4–350.1	133.02	5.92 × 10^7^	0.99
C-KCl	289.2–350.9	126.02	1.91 × 10^9^	0.99
C-CaCl_2_	290.4–350.4	154.93	3.67 × 107	0.98
C-MgCl_2_	293.1–350.7	157.67	2.89 × 10^8^	0.99
C-CH_3_COONa	280.7–349.8	79.61	1.23 × 10^3^	0.99
C-CH_3_COOK	279.9–350.7	71.15	3.61 × 10^3^	0.97
C-(CH_3_COO)_2_Ca	304.8–355.9	148.40	6.62 × 10^9^	0.99
C-(CH_3_COO)_2_Mg	300.1–350.2	143.01	5.79 × 10^9^	0.99

## Data Availability

The original contributions presented in this study are included in the article. Further inquiries can be directed to the corresponding author.
